# Hybridization between char species (*Salvelinus alpinus* and *Salvelinus fontinalis*): a fast track for novel allometric trajectories

**DOI:** 10.1242/bio.033332

**Published:** 2018-10-15

**Authors:** Bernard-Antonin Dupont Cyr, France Dufresne, Felix Christen, Véronique Desrosiers, Émilie Proulx, Nathalie R. Le François, Grant W. Vandenberg, Pierre U. Blier

**Affiliations:** 1 Département de biologie, chimie et géographie, Université du Québec à Rimouski, 300, Allée des Ursulines, Rimouski, QC, Canada G5L 3A1; 2 Faculté des sciences de l'agriculture et de l'alimentation, Département des sciences animales, Université Laval, Pavillon Paul-Comtois 2425 Rue de l'agriculture Local 1122, Québec, Québec, Canada, G1V 0A6; 3 Biodôme de Montréal, 4777 Avenue Pierre-De Coubertin, Montréal, Québec, Canada H1V 1B3

**Keywords:** Arctic char, Brook char, Ontogeny, Morphometry, Heterosis, Transgressive segregation

## Abstract

Hybridization between closely related species can generate genetic and phenotypic variation, providing valuable biological material to assess the physiological impact of the structural or functional variability of different organs. In the present study, we examined growth rates of various organs and whole body in brook char, Arctic char and their reciprocal hybrids over a period of 281 days. Parental species achieved significantly higher body mass than their hybrids. Hybridization significantly reduced the relative size of the heart, liver and spleen. The relative size of pyloric caeca did not differ among the four groups. The observed lower growth performance of the hybrids compared to parental species strongly suggests that divergence in the relative size of digestive organs, liver and heart partly dictate growth capacity. Our results also suggest that the increased variability achieved through hybridization may prove useful in a genetic selection program.

## Abstract (precis)

**Summary:** This research provides a comprehensive overview of the phenotypic modifications in growth trajectories of body size and internal organs following interspecific hybridization in chars.

## INTRODUCTION

Hybridization can lead to rapid genomic changes, including chromosomal rearrangement, genome expansion, differential gene expression and gene silencing (Hoffmann and Sgrò, 2011; Josefsson et al., 2006; Morales and Dujon, 2012; Otto, 2007; Tirosh et al., 2006). First generation hybrids harbor a genetic combination of both parental species, which could result in new and different ontogenetic trajectories that could produce phenotype novelties (Corse et al., 2012). Heterosis arises from the combination of superior alleles at multiple loci, allelic interactions with one or multiple hybrid alleles and epistasis (Hochholdinger and Hoecker, 2007) and often results in the expression of superior traits in first-generation hybrids compared to parental lines. Research on salmonids has however shown that first-generation hybrids often perform less well than parental lines (Bartley et al., 2000; Bryden et al., 2004; Miller et al., 2004), suggesting that disruption of additive effects and dominance interactions can potentially reduce growth performance in first-generation hybrids (McClelland et al., 2005).

Arctic char (*Salvelinus alpinus*) and brook char (*Salvelinus* *fontinalis*) are two freshwater char species that recolonized eastern Canada following the last glaciation. Both species are qualified as generalists (insectivory and piscivory) but are known to exhibit different polymorphisms (benthic and pelagic morphs) and different ecological specialization (landlocked, anadromous) (Bertrand et al., 2008; Jonsson and Jonsson, 2001; Morinville and Rasmussen, 2008; Woods et al., 2013). Their habitat preferences are mainly characterized by temperature; Arctic char is a northern species acclimated to harsh cold water (5–19°C), while brook char prefers warmer water (8–20°C) (Larsson, 2005; Peterson et al., 1979; Sutterlin and Stevens, 1992). Their geographical distribution thus overlaps and hybrids between *S. alpinus*×*S. fontinalis* are known to be viable in the laboratory (Dumas et al., 1992) and have also been reported in the wild (Bernatchez et al., 1995; Glemet et al., 1998; Hammar et al., 1991). Current climatic fluctuations may modify their distribution area and at the same time, increase cohabitation and hybridization propensities. Theoretically, this situation could stimulate the emergence of hybrid individuals with distinct phenotypes, which could give them survival or fitness advantage.

In char, most of the work on hybridization has focused on the growth of first-generation hybrids (Dumas et al., 1992, 1996) and has shown that F1 hybrids generally display mid or lower growth performance than parental species. However, few studies have examined the underlying physiological causes associated with low growth performance of hybrids. Previous work on the Atlantic cod (*Gadus morhua*) and salmonid species have revealed that growth and digestive performance are linked to the development of digestive organs like the digestive tract and pyloric caeca (Blier et al., 2002, 2007; Lemieux et al., 1999, 2003; Stevens and Devlin, 2000). Since hybridization can impair the rate or timing of developmental processes and lead to allometric differences between parents and their offspring, we suggest that hybridization may affect the ontogenic trajectory of digestive organs, which in turn could change digestive capacities and growth performance. To better understand the consequences of hybridization, it is necessary to understand the ontogenic trajectories of the main physiological organs that constrain growth performance and interfere with development.

This study aimed to examine organ and body growth in first-generation hybrids from two char species from North American populations: brook char (*S. fontinalis* Baldwin; BC) and Arctic char (*S. Alpinus* Fraser; AC) (female Arctic char×male book char, HA; female brook char×male Arctic char, HB). We hypothesized that hybridization will increase phenotypic variability and cause modifications/adjustments of organ ontogenic trajectories in reciprocal hybrids in comparison to the parental species. These phenotypical novelties could be of great significance considering the functional link between digestive capacity, physiological trade-off and growth performance.

## RESULTS

**Fig. 1.**
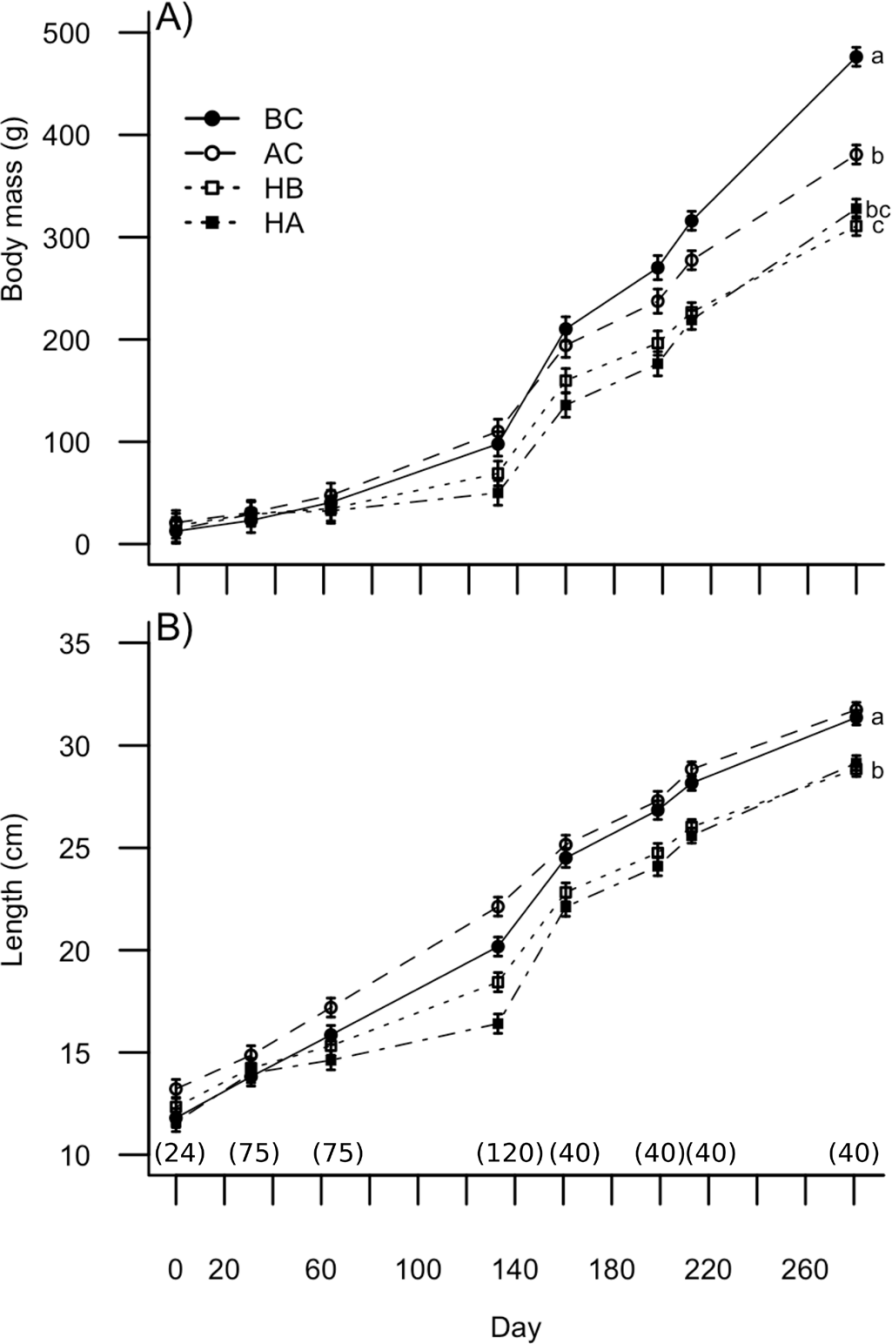
Body mass (A) and total length (B) of brook char (BC), Arctic char (AC), hybrid female Arctic char (HA) and hybrid female brook char (HB) in relation with day. Linear mixed effect model suggested a significant difference (*P*<0.05) between groups for body mass and length at day 64, 139, 190, 200 and 281 and were indicated with a different letter. Sample size for each sampling experimental group at every sampling period was given as number in a parenthesis. Results are given in mean±s.e.m.

Body mass (17.1±1.6 g; *P*=0.068) and length (12.3±0.4 cm; 0.196) were similar between groups at the start of the experiment. Body mass and length became significantly different between groups on day 64 and these differences were maintained for the remainder of the growth trials (Fig. 1). After 281 days, BC body mass (475.5±25.6 g) was 19.7% higher than AC (381.8±15.5 g) and 32.8% higher than in both hybrids (HA 327.7±34.5 g; HA 311.6±29.0 g) (Fig. 1A). Parental AC (31.8±0.4 cm) and BC (31.4±0.5 cm) were 8.2% longer than in both hybrids (HA 29.1±1.0 cm; HB 28.9±0.9 cm) (Fig. 1B). Body mass and length allometry were significantly different (*P*<0.001) between parental species (AC=BC; R^2^=0.973; y=0.004x^3.298^) and their reciprocal hybrids (HA=HB; R^2^=0.980; y=0.005x^3.235^).

**Fig. 2.**
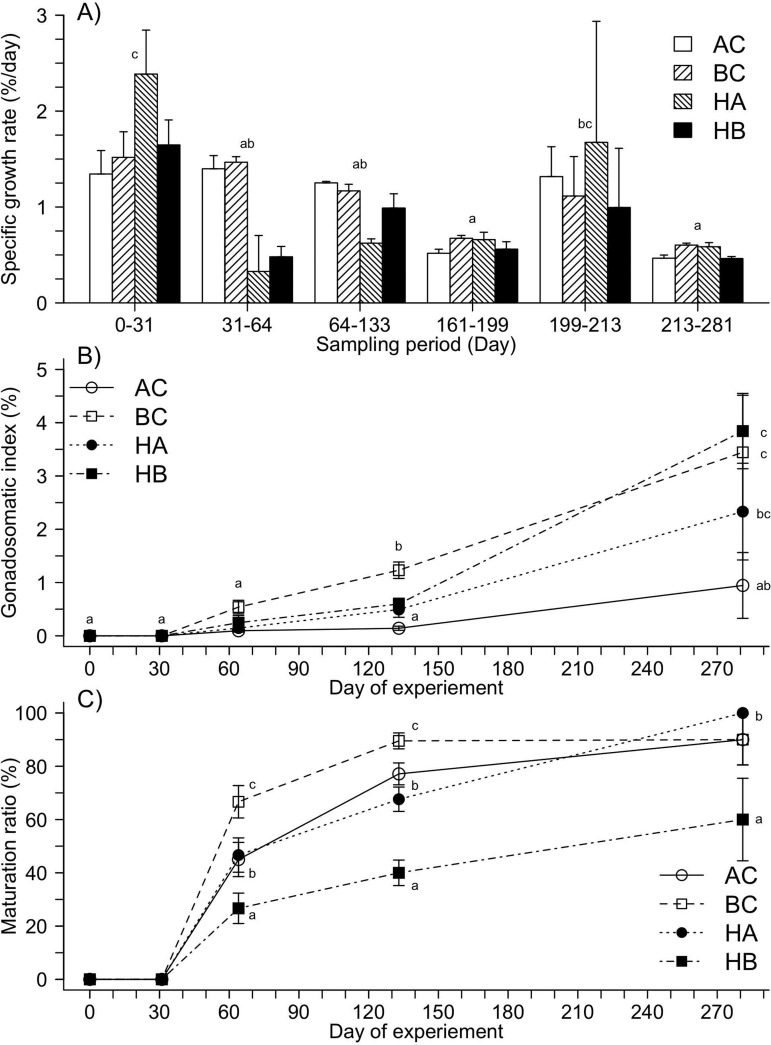
(A) Specific growth rate, (B) gonado-somatic index and (C) maturation ratio (%) of brook char (BC), Arctic char (AC) hybrid female Arctic char (HA) and hybrid female brook char (HB) in relation with day. Maturation ratio corresponds to the percentage of sexable fish. Results are given in mean±s.e.m. In specific growth rate figure (A) significant differences between sampling periods were indicated with a difference letter, while different letters in gonado-somatic index figure (B) indicates a significant difference between the day of experiment and genotype.

Despite these differences in final body mass and length, mean calculated SGR did not differ among groups (0.95±0.06% day^−1^) (*P*=0.522). Specific growth rate (SGR) significantly decreased in relation with body mass (ANVOCA; *P*<0.001, R^2^=0.348, y=3.549x^−0.138^) with no significant group effect (*P*=0.185). For all groups, SGR were highest in the first 31 days, decreased between days 31 to 199, increased over days 199 to 213 with the onset of gonadal development and then decreased on days 213 to 281 (Fig. 2A). The first signs of gonadal development were observed on day 64 at 13 months post-hatching, after which time the frequency of matured fish significantly increased (*P*<0.01) until day 281 (Fig. 2B and C). Gonado-somatic index (GSI) was similar among groups during the first 64 days of the experiment. On day 133, GSI was significantly higher in BC than in other groups (Fig. 2B). On day 64, GSI was significantly higher for males compared to females (*P*<0.001), but female GSI was highly variable among individuals and the difference between the sexes was no longer apparent on day 281 (*P*=0.832). GSI was significantly different among groups (males and females together) on day 281. Brook char (3.4±1.1%) and hybrids (HA 2.3±0.9%; HB 3.8±0.7%) displayed a higher GSI than AC. There was a significant interaction of maturation proportion, days of experiment and groups (*P*<0.001; Fig. 2C). After 281 days, the proportion of matured fish was lower for HB than other groups (Fig. 2C).

During the experiment, the relative size of the heart, the pyloric caeca and the intestine decreased significantly (*P*<0.001). AC cardio-somatic index was 22.4% higher than HB and 30.5% higher than BC and HA (*P*<0.001) (Fig. S1A). Accordingly, final cardio-somatic indices were 0.105±0.005, 0.083±0.003, 0.101±0.005 and 0.100±0.005% for AC, BC, HA and HB respectively. Hepatic somatic indices changed marginally during the experiments (R^2^<0.03; Fig. S1B) and the amplitude of these changes varied depending on the group (*P*=0.007). No statistically significant differences in spleen somatic index were found among the different groups (0.114±0.014%) (*P*=0.166). The relative size of digestive organs (pyloric caeca and intestine) decreased significantly over the course of the experiment (*P*<0.001). Arctic char (0.451±0.043%), BC (0.633±0.038%), HA (0.601±0.037) and HB (0.412±0.049) final intestine somatic indices were significantly different from each other (*P*=0.005) while final pyloric caeca somatic indices were similar (*P*=0.144) among groups (1.24±1.0%). However, ANCOVA analyses revealed interaction between group and experiment day for somatic indices of pyloric caeca somatic indices (*P*=0.001; Fig. S1D) and intestine (*P*<0.001, Fig. S1E) that decreases over time. The same trend was observed for length and all somatic index (results not shown).

**Fig. 3.**
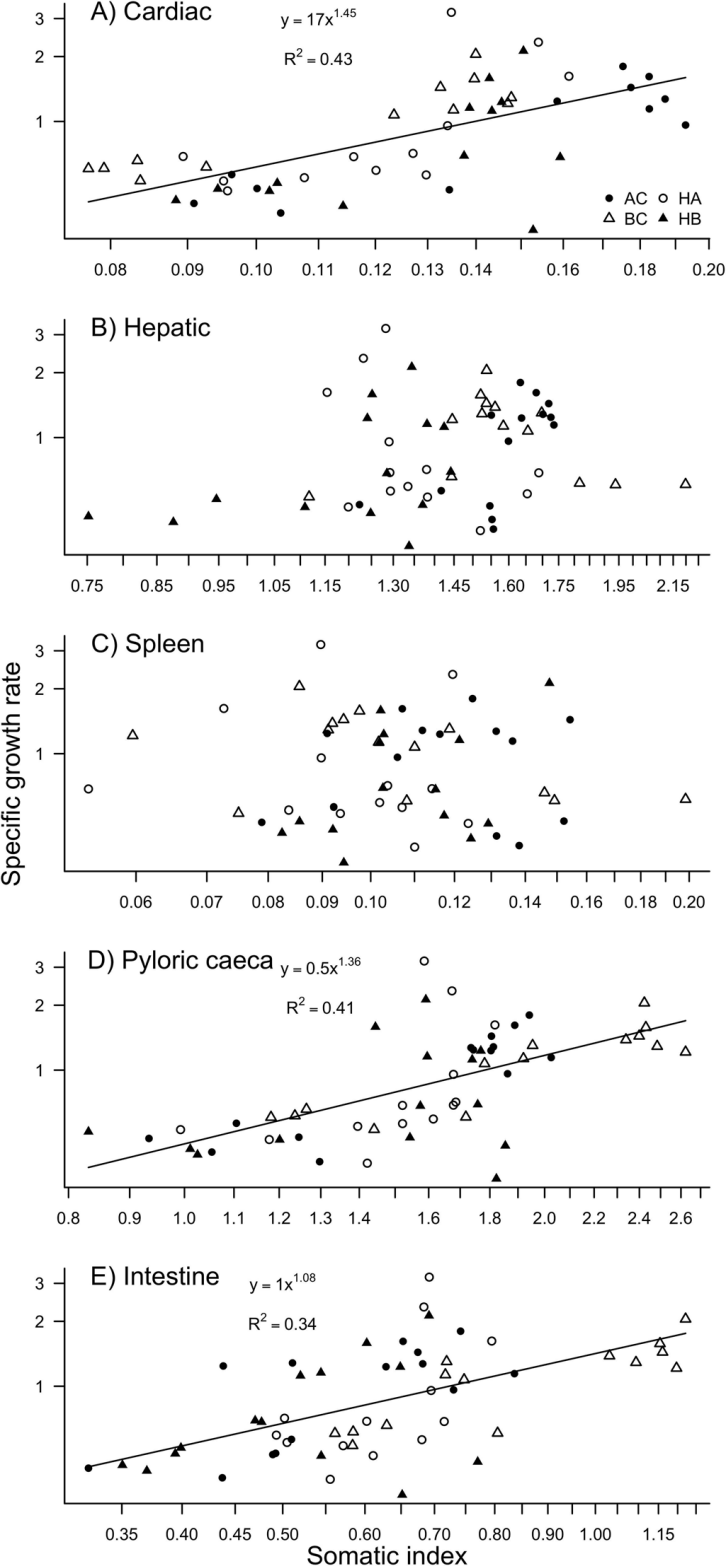
Relationship between specific growth rate and (A) cardiac, (B) hepatic, (C) spleen, (D) pyloric caeca or (E) intestine somatic of Arctic char (AC), brook char (BC), hybrid female Arctic char (HA) and hybrid female brook char (HB).

Specific growth rate was positively correlated with cardiac (*P*<0.001), pyloric caeca (*P*<0.001) and intestines (*P*<0.001) somatic indices (Fig. 3). No relationship was observed between SGR and hepatic (*P*=0.125) or spleen (*P*=0.881) somatic indices. For all regression between SGR and somatic index, no significant effects of the group or interaction between group and somatic indices were found.

**Fig. 4.**
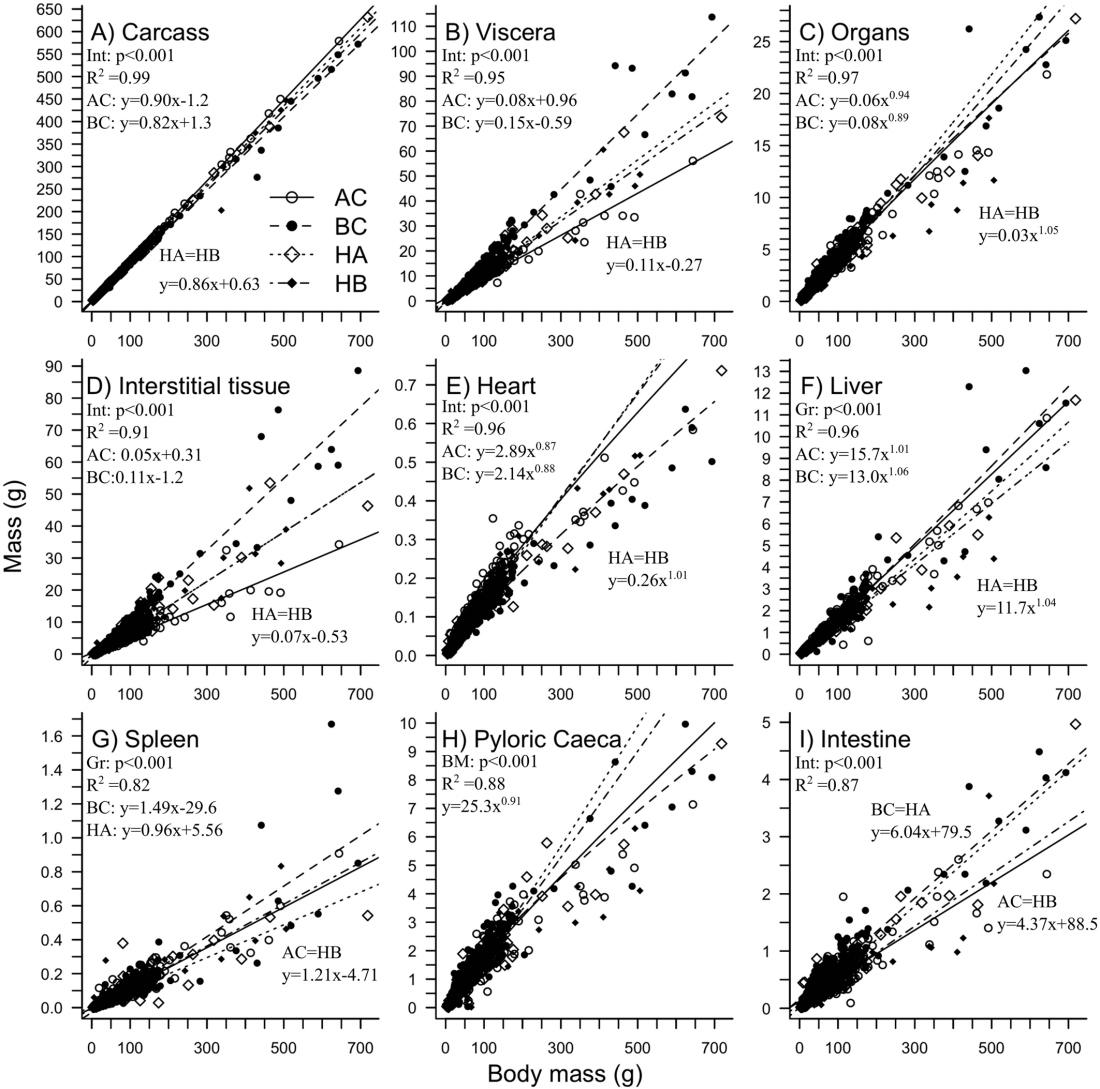
Relationships between carcass (A), viscera (B), total organs (C), abdominal interstitial tissues (D), heart (E), liver (F), spleen (G), pyloric caeca (H), intestine (I) and the body mass of brook char (BC), Arctic char (AC), hybrid female Arctic char (HA) and hybrid female brook char (HB). Carcass, viscera, remaining visceral, spleen and intestine allometric model was expressed with a linear model (y=mx+b) while total organs, heart, liver and pyloric caeca allometric model was expressed with a logarithmic model (y=ax^k^). Coefficient for carcass, viscera, organs and abdominal interstitial tissues are in g per g of body weight, while heart, liver, spleen, pyloric caeca and intestines are in mg per g of body mass. *P*-values were BM, body mass; Gr, groups and Int, Interaction.

According to ANCOVA analysis, the allometric slopes were significantly different among groups for carcass, viscera, abdominal interstitial tissue and intestine, whereas spleen elevations were significantly different between groups (Fig. 4). The relationship between carcass and body mass (Fig. 4A) suggests that AC carcass mass grew 4.3% faster than hybrid carcasses and 8.8% faster than BC, with hybrid carcasses having an intermediary growth trajectory (AC>[HA=HB]>BC). The viscera–body mass relationship was the exact reverse of the carcass (BC>[HA=HB]>AC) (Fig. 4B). Organ mass relationship were significantly different in hybrid than in parental ([HA=HB]>[AC=BC]) (Fig. 4C). The major difference in body mass can be attributed to the abdominal interstitial tissues (AC<[HA=HB]<BC) (Fig. 4D). AC had the lowest mass of abdominal interstitial tissues, hybrid displayed intermediate values and BC had larger abdominal interstitial tissue mass. Hybridization considerably modified heart, liver, spleen and intestine allometries. Increases in heart mass relative to body mass were higher in hybrids compared to parental strains, particularly when compared to BC (Fig. 4E). BC had the highest increment in spleen relative mass (Fig. 4G). Hybridization affected the mass of the liver ([HA=HB]<BC<AC]) (Fig. 4F) and the intestine ([AC=HA]<[BC=HB]) (Fig. 4I). Pyloric caeca allometry was highly variable and did not differ among groups (Fig. 4H).

## DISCUSSION

First generation hybrids often show higher growth rates than parental lines due to heterosis (Fjalestad, 2005). By contrast, our results showed that hybridization between brook and Arctic chars led to lower final mass and length, suggesting hybrid breakdown (Harrison and Burton, 2006; Presgraves, 2003). Our results are at odds with those of Dumas et al. (1996), who reported intermediate parental body weight in hybrids between AC and BC (but from different strains). A lack of heterosis appears prevalent in numerous salmonid species. Lower interstrain hybrid growth rates have been observed in rainbow trout (Blanc et al., 2000; Tymchuk et al., 2007), Atlantic salmon (Gjerde and Refstie, 1984), Chinook salmon (Bryden et al., 2004), in pink salmon (Gilk et al., 2004) and in coho salmon (McClelland et al., 2005). Blanc and Chevassus (1982) noticed the same trend in interspecific crossbreeding between coho×chinook salmon, brown×brook trout and rainbow trout×coho salmon. Fish were raised at 10–11°C, which is in the range of optimal temperature for both species (Arctic char ranges 5–19°C and Brook char ranges 8–20°C) (Beitinger et al., 2000; Elliott, 1982). This temperature range was therefore close to the optimal for all groups, which was reflected by a low mortality rate. It is, however, possible that hybridization induced a shift in temperature optimum, resulting in a significant impact on organ and body growth patterns.

Transgressive segregation is another outcome of hybridization and is defined by the appearance of a hybrid extreme phenotype relative to their parental phenotype (Albertson and Kocher, 2005). Hybrids HA and HB had smaller final body weight and length than both parental species, which represent an extreme negative phenotype (Pereira et al., 2014). Hybridization can also lead to unpredictable phenotype variability that can be further modified by selection. Fast growth might not necessarily be the most profitable phenotype and might mask a possible trade-off between different organs and carcass growth. Body mass can be divided into two major components: the carcass and the viscera. AC had the highest carcass ratio, BC had the highest viscera ratio and hybrids had intermediate ratios. These differences were associated with modifications of fish allometry. Previous studies have shown that eviscerated carcass ratio varies substantially between AC and BC (Miglavs and Jobling, 1989). Whole viscera mass was variable among parental groups but total organ mass was not. The main differences in viscera composition were mostly dictated by the abdominal interstitial tissues (fatty tissues and gonads). BC appear to invest more in abdominal interstitial tissues than the other groups. Fat and gonads are the principal abdominal interstitial tissue component and BC is well known to invest more resources in both of them (Miglavs and Jobling, 1989). Optimal design might require a trade-off among system components that could be restricted by metabolism and energy demand. The relative size between high maintenance cost organs such as brain, heart, liver, digestive system, gonads and kidney needs to be in balance with the relative size of less consuming organs such as muscle, bones, fat and other structures (Rosenfeld et al., 2015). Body parts structure optimization might respond to the required balance between the functions of the tissues and their maintenance cost. This balance can be driven by adaptive environmental flexibility and their metabolic strategies (Armstrong and Schindler, 2011). In rainbow trout (Rasmussen and Ostenfeld, 2000; Weatherley and Gill, 1983) and Atlantic salmon (Wathne, 1995), fast-growing fish tend to invest in low-consumption viscera deposition (i.e. abdominal fat). Our fastest growing group, BC, follows the same strategy as they displayed the highest abdominal interstitial tissues content.

Individual growth rates were correlated to heart and digestive organs’ relative mass when the data from the four groups were combined, suggesting that increased digestive and cardiac capacities are associated with higher growth rates. The relative size of the heart cannot, however, explain the observed growth divergences among groups since AC, that has the highest relative heart size, reached a lower body mass than BC at the end of experiment. The relative size of the pyloric caecae can neither explain the higher body mass of BC since HA and HB had the highest increase in pyloric caecae relative to body mass but reached the lowest final body size.

Digestive capacity and, specifically, the activity of proteolytic enzymes, has been identified as a potential factor limiting fish growth (Bélanger et al., 2002; Blier et al., 1997; Lemieux et al., 1999). Lemieux et al. (1999) reported the presence of a positive correlation between growth rates and pancreatic trypsin activity in Atlantic cod (*G.* *morhua*). Bergot et al. (1981) and Blier et al. (2002) proposed that salmonid growth could be correlated to the size of the digestive organs or to the number of pyloric caeca appendices as it increases the contact area (food assimilation and increased digestibility) as well as the overall capacity of the organ to synthesize digestive enzymes. Blier et al. (2002), Stevens and Devlin (2000) and Stevens et al. (1999) have shown that transgenic salmon possess enhanced gut surface areas, which suggests that their enhanced growth may partly be the result of a larger intestinal size. In our study, pyloric caecum allometry slopes were not significantly different among groups. The absence of a clear allometry divergence between groups might suggest that activities of digestive tissues, for example the rate of production of proteolytic enzymes in pyloric caeca, could be more of a determinant for setting growth rate than the relative mass of the tissues.

From a breeding perspective, Arctic char's higher carcass growth seems more alluring than the BC's high visceral growth phenotype. By evaluating hybrids intermediate carcasses and viscera allometry, we would have expected an intermediate growth trajectory, which is not what we observed. In the present study, organ allometry was significantly remodeled in the different groups. Total organ mass of both parental lines showed similar growth trajectory and allometry but body components were significantly different between AC and BC. More importantly, none of the hybrid lines followed a specific organ parental trajectory. Hybridization resulted in organ allometry remodeling and hybrid body-part structures followed their own ontogeny trajectory.

Our study revealed that hybridization led to the production of divergent phenotypes with different developmental trajectory of internal organs, when compared to parental lines. These divergent phenotypes can be highly valuable in aquaculture for designing optimal phenotypes through selective process, or as a model to better understand the physiological modulator of digestive capacity and growth process.

## MATERIALS AND METHODS

### Fish, facilities and experimental design

The experiment was conducted on two parental species: Arctic char and brook char and hybrids with Arctic char as mothers and with brook char as mothers. 600 10 g (10 months post-hatching) fishes of each type were supplied by Pisciculture des Monts de Bellechasse Inc. (Saint-Damien-de-Buckland, Canada) and Aquaculture Gaspésie Inc. (Gaspé, Canada). Growth trials were conducted at the Laboratoire de recherche en sciences aquatiques (LARSA; Université Laval, Québec, Canada). Fish were randomly stocked in 12 0.150 m^3^ tanks supplied with 99% of recirculating freshwater set at 10.5°C, dissolved oxygen was set at 9.7 mg/ml and photoperiod was 16L:8D. Water quality was monitored daily and fish were submitted to a 2-month acclimation period and fed a ration of 1% of their average body weight for the first month and hand-fed to satiety in the second month. The experimental protocol was performed in accordance with the Good Animal Practice certificate issued by the Canadian Council on Animal Care (CCAC, Ottawa, Canada).

### Feed and feeding

During the experimental period, fish were fed on a 7 day schedule protocol. For the first 2 days fish were hand-fed to satiety twice a day, then on the next 4 days fish received two meals consisting of 80% of the average food intake during the first 2 days. Fish were fasted on the last day. Diet formulation is shown in Table 1. Diets were mixed then steam pelleted using a California Pellet Mill (Model CPM CL-5, Crawfordsville, USA), dried overnight in a forced-air oven set at 22°C and thereafter stored at 4°C. Pellet size was adjusted to fish size throughout the experiment.

**Table 1.**
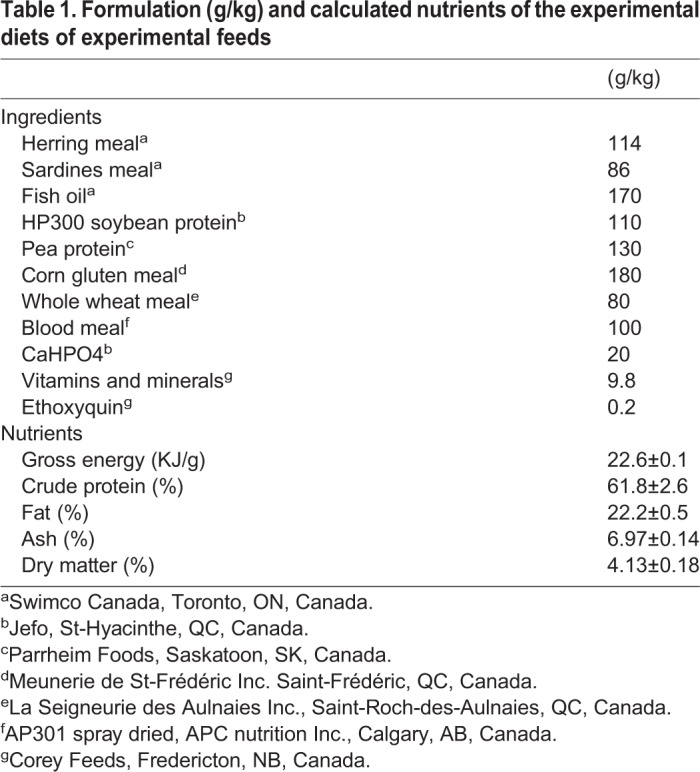
**Formulation (g/kg) and calculated nutrients of the experimental diets of experimental feeds**

### Sample and measurements

The growth trial was conducted over 281 days. Fish were fasted for 3 days prior to any manipulation or sampling activities and anesthetized using an 80 ppm MS-222 solution (Aqualife MS 222, Syndel Laboratory Inc., Nainamo, Canada). Total length and wet mass were measured monthly. At days 0, 31, 64, 133 and 281, 15 fish per tank were euthanized with a blow behind the head. For each individual, carcass, viscera and organs were dissected and weighed. Visceral mass was measured by weighing all the abdominal organs which included pyloric caeca, intestines, heart, liver, spleen, gonads, swim bladder, as well as abdominal fat deposits. SGR was evaluated monthly according to the formula from Jobling (1983):

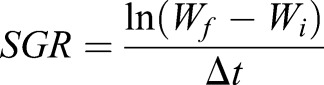
SGR is expressed in percentage day^−1^, *W_i_* is fish body mass at an initial time, *W_f_* is fish body mass at a final time and *t* is duration in days. Total organ mass was calculated by summation of the heart, liver, spleen, pyloric caeca and intestine mass. Abdominal interstitial tissue mass was calculated as the difference between viscera mass and total organs mass; abdominal interstitial tissues included abdominal fatty tissues and gonads. For each organ, a somatic index was evaluated as their relative mass. Maturation ratio was defined as the percentage of fish demonstrating identifiable gonads.

### Nutrients analysis

Nutrients analysis was performed on each batch of aquafeed produced (*n*=20). Gross energy was evaluated with a Parr 6100 calorimeter (calorimeter, Parr Instrument Company Inc., Moline, USA). Crude protein was measured using CN analysis performed on Costech 4010 (Costech Analytical technologies Inc., Valencia, USA). Quantification was based on in-run acetanilide standard (Fluka, Honeywell Research Chemicals Inc., Mexico City, Mexico) with calibration range of 0.025 to 0.080 mg and 0.170 to 0.550 mg for nitrogen and carbon respectively. Aquafeed lipid content was determined by Soxhlet extraction (24 h) using chloroform: ethanol (2:1; v:v) as a solvent. Ash content was measured by dry ashing in porcelain crucibles in a muffle furnace at 550°C overnight (12 h). Sample weight was recorded before drying and after a few hours of cooling in the oven. Dry matter was measured by drying samples at 80°C and water content was evaluated when samples reached a stable mass (24 h).

### Statistical analysis

Data were analyzed with R software (R Foundation for Statistical Computing, Vienna, Austria). Normality of residuals and equality of variance were estimated with Shapiro test and Levene test respectively. Body mass and length were analyzed with a linear mixed effect model (LMEM) using group and sampling day as fixed effect, while random effect was attributed to rearing tank. SGR and GSI were analyzed with a two-way analysis of variance (ANOVA) using sampling period and groups as factors. Somatic index was analyzed with a one-way ANOVA using group as factors. LMEM and ANOVA were accompanied by Tukey's highly significant difference multiple comparison test.

Ontogenic trajectory of parental species and reciprocal hybrids were compared to detect heterochronic changes. We compared the carcass, abdominal interstitial tissues, visceral and organ mass allometric trajectories to test the presence of acceleration/neoteny or predisplacement/postdisplacement (McKinney, 1986). As for data distribution, the allometric model was expressed as y=ax^k^ or as y=mx+b when appropriate. For each ANCOVA a random effect was attributed to the rearing tank. Significant differences of the scaling exponent (k) or the slope (m) were evaluated using contrast and significant difference of the shape coefficient (a) or intercept (b) was evaluated using Tukey's post hoc. Significance levels were *P*<0.05 and results are expressed as mean with the standard error (s.e.m.).

## Supplementary Material

Supplementary information
