# Supplementary material for: Hybridization between char species (Salvelinus alpinus and Salvelinus fontinalis): a fast track for novel allometric trajectories
Source: Biol Open. 2018 Oct 15;7(10):bio033332. doi: 10.1242/bio.033332 (PMC6215407; doi:10.1242/bio.033332)
Supplement: Supplementary information [file biolopen-7-033332-s1.pdf]

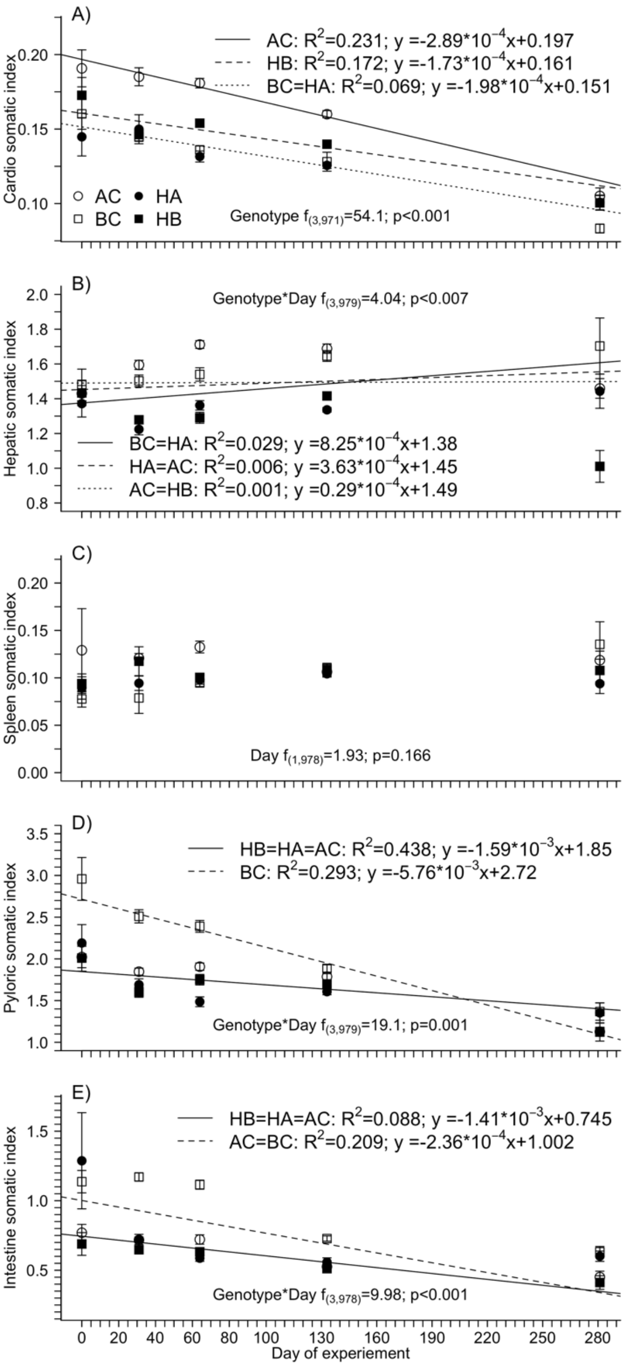

Figure S1: Relationship between days of experiment and somatic index of Arctic char (AC), brook char (BC), hybrid female Arctic char (HA) and hybrid female brook char (HB). A) cardio, B) hepatic, C) spleen, D) pyloric and E) intestine somatic.
